# Empyema associated with *Actinomyces georgiae* successfully treated with multimodal therapy including surgical intervention: A first case report

**DOI:** 10.1016/j.rmcr.2025.102254

**Published:** 2025-07-02

**Authors:** Senichi Fukuda, Yuya Homma, Hiroki Kawakami, Soichiro Yamaji, Yuki Sato, Nao Sato, Reina Idemitsu, Taiki Kawai, Naoki Inoshima, Jun Hayashi, Norihiko Kubota, Tatsuya Nagai, Ayumu Otsuki, Hiroyuki Ito, Hiroshi Sugimura, Kei Nakashima

**Affiliations:** a Department of Pulmonology, Kameda Medical Center, 929 Higashi-cho, Kamogawa, Chiba, 296-8602, Japan; b Department of Thoracic Surgery, Kameda Medical Center, 929 Higashi-cho, Kamogawa, Chiba, 296-8602, Japan

**Keywords:** Actinomycosis, Empyema, *Actinomyces georgiae*, Video-assisted thoracoscopic surgery

## Abstract

A 77-year-old man presented with right-sided chest pain and dyspnea. Computed tomography revealed a loculated pleural effusion, and thoracentesis yielded purulent fluid. *Actinomyces georgiae* was identified using pleural fluid culture. Despite treatment with ampicillin-sulbactam and thoracic drainage, the patient's condition worsened, requiring video-assisted thoracoscopic surgery. The patient recovered completely after completing an 11-month course of antibiotics. This is the first reported case of empyema associated with *A. georgiae*. *Actinomyces* should be considered as a potential cause of empyema, and a comprehensive approach, including surgical intervention, is necessary for optimal management.

## Highlights

• First report of empyema associated with *Actinomyces georgiae.*
• Failed initial antibiotics/drainage for *A. georgiae* empyema.
• Required VATS decortication and prolonged (11-month) antibiotic therapy.
• *Actinomyces* empyema often requires surgical intervention for resolution.
• Consider *Actinomyces* in empyema; long-term antibiotics are essential.

## 1 Introduction

Actinomycosis is a chronic granulomatous infection caused by *Actinomyces* species, which are branching, Gram-positive anaerobic bacteria typically found as commensals in the oropharynx, intestinal tract, and female genital tract [1]. Although most infections occur in the cervical-oral region (50–70 %), the pulmonary region accounts for 15–20 % of cases [2,3]. Pulmonary actinomycosis typically presents with radiographic findings that mimic lung cancer, alongside nonspecific symptoms, such as cough, sputum, hemoptysis, chest pain, dyspnea, fever, weight loss, malaise, and night sweats, complicating diagnosis [1]. The initial diagnostic accuracy for pulmonary actinomycosis can be as low as 4 %, with frequent misdiagnosis as lung cancer [4]. At least 49 *Actinomyces* species have been identified, with more than 26 associated with human disease [5]. Human actinomycosis is primarily caused by *A. israelii*, while *A. graevenitzii* is the most commonly associated with pulmonary actinomycosis [6]. Thoracic infections caused by *Actinomyces* are rare [6]. Although empyema has been reported in infections with *A. meyeri, A. israelii, A. odontolyticus, A. naeslundii*, *A. turicensis*, and *A. viscosus*, to our knowledge, there are no reported cases of empyema caused by *A. georgiae* [[7], [8], [9], [10], [11], [12]].

We present a case of polymicrobial empyema in which *A. georgiae* was identified. The patient did not improve with antibiotics and chest drainage but showed response to combined modality treatment including surgical intervention.

## 2 Case

A 77-year-old man presented to a primary care physician with a 12-day history of right lateral chest pain and dyspnea on exertion. A chest X-ray revealed an infiltrative shadow in the right lung, leading to a diagnosis of pneumonia and a referral to our hospital. His medical history included chronic kidney disease, diabetes mellitus, and hypertension. He reported a history of smoking in his twenties but had quit, with no history of allergies.

His vital signs were as follows: blood pressure 141/91 mmHg, heart rate 100 beats/min, respiratory rate 20 breaths/min, oxygen saturation 94 % on room air, and body temperature 36.6 °C.

**Fig. 1:**
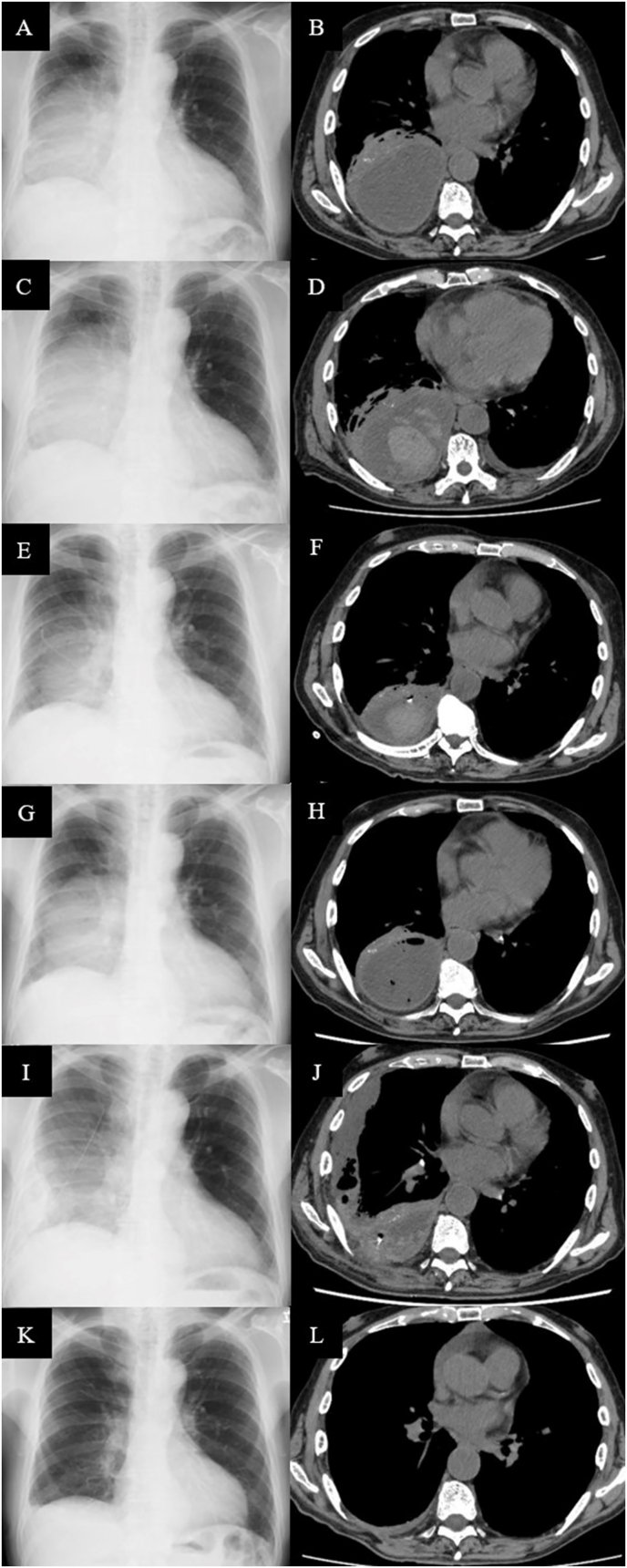
Imaging results of a 77-year-old man with *Actinomyces* empyema. On admission, chest X-ray revealed opacities in the right middle and lower lung fields (A), and computed tomography (CT) scan showed a loculated pleural effusion in the right lung (B). On day 4, chest X-ray displayed persistent opacities in the right middle and lower lung fields (C). CT imaging revealed a high-density area suggestive of hemorrhage, leading to the decision to withhold urokinase administration (D). Following antibiotic therapy and drainage, chest X-ray showed improvement in opacities (E), and CT scan revealed a reduction in pus accumulation (F). However, after the patient developed fever on day 17, chest X-ray showed increased opacities in the right lung (G), and CT scan indicated worsening pleural effusion (H). Following video-assisted thoracoscopic surgery (VATS), chest X-ray demonstrated improvement in the right lung opacities (I), and CT scan confirmed further reduction in pus accumulation (J). Complete resolution of the empyema was confirmed after an 11-month course of antibiotics (K, L).

Physical examination was notable only for diminished breath sounds over the right posterior lung field. Laboratory tests revealed anemia (7.9 g/dL), leukocytosis (15,200/μL), thrombocytosis (52.0 × 10^4^/μL), and elevated levels of C-reactive protein, creatinine, blood urea nitrogen, aspartate aminotransferase, alkaline phosphatase, and γ-glutamyltransferase (Table 1). A chest X-ray showed opacities in the right middle and lower lung fields (Fig. 1A), while computed tomography (CT) revealed a loculated pleural effusion in the right lung, compressing the right lower lobe (Fig. 1B). Thoracentesis was performed, yielding purulent pleural fluid with a low glucose level (2 mg/dL), elevated lactate dehydrogenase (34,254 U/L), and elevated total protein (4900 mg/dL). Gram staining of the pleural fluid specimen showed elongated Gram-positive rods, small Gram-positive streptococci, and small Gram-negative rods. Based on these clinical findings, empyema was diagnosed, and the patient was admitted for thoracic drainage.

**Table 1:** Laboratory findings at the time of first admission.

Hematology			Biochemistry			Pleural effusion		
White blood cells	15200	/μL	Total protein	6.6	g/dL	Appearance	Purulent	
Neutrophils	85.6	%	Albumin	2.0	g/dL	Cell count	108800	/μL
Lymphocytes	8.5	%	Total bilirubin	0.4	mg/dL	Neutrophils	99	%
Monocytes	5.7	%	Aspartate aminotransferase	48	U/L	Lymphocytes	0	%
Eosinophils	0.1	%	Alanine aminotransferase	31	U/L	Others	0	%
Basophils	0.1	%	Lactate dehydrogenase	166	U/L	Macrophages	1	%
Red blood cells	297 × 10^4^	/μL	Alkaline phosphatase	277	U/L	Glucose	2	mg/dL
Hemoglobin	7.9	g/dL	γ-glutamyltransferase	65	U/L	Protein	4900	mg/dL
Hematocrit	25.0	%	Blood urea nitrogen	27	mg/dL	Albumin	1500	mg/dL
Platelets	52.0 × 10^4^	/μL	Creatinine	2.72	mg/dL	Lactate dehydrogenase	34254	U/L
			Creatine kinase	97	U/L			
			C-reactive protein	17.98	mg/dL			
			Na	139	mEq/L			
			K	4.0	mEq/L			
			Cl	106	mEq/L			
			Glucose	158	mg/dL

Empirical treatment with ampicillin/sulbactam (3.0 g IV every 12 hours) was initiated. A pleural effusion on the right dorsal side made bedside chest tube insertion challenging, prompting elective CT-guided thoracic drainage. CT-guided drainage procedures are not scheduled on weekends or public holidays in our hospital unless deemed critically urgent. The patient was admitted on Saturday, but the subsequent Monday was a public holiday, and hence the procedure was performed on day 4. Chest radiography on day 4 revealed opacities in the right middle lung field (Fig. 1C). Although urokinase administration was initially considered, CT imaging revealed a high-density area suggestive of hemorrhage (Fig. 1D), leading to its discontinuation. Pleural fluid culture revealed *Parvimonas micra*, *Actinomyces georgiae* and *Fusobacterium* species, identified at the species-level by Matrix-assisted laser desorption ionization-time of flight mass spectrometry (MALDI-TOF MS). The patient continued to receive ampicillin/sulbactam as the isolated bacteria were susceptible to antibiotic. Cavities were suspected as entry points for the infection, and dental extraction was recommended. However, the patient did not consent to the procedure, and oral care was provided. X-ray on day 10 and CT on day 11 improved (Fig. 1E and F), and the chest drain was removed on day 12 due to a substantial reduction in drainage. On day 17, the patient developed fever, and chest X-ray showed increased opacities in the right lung (Fig. 1G). A subsequent CT scan revealed worsening pleural effusion (Fig. 1H).

**Fig. 2:**
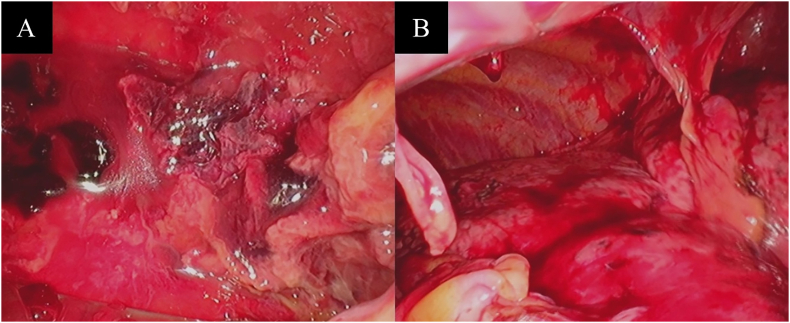
Intraoperative view of video-assisted thoracoscopic surgery before (A) and after (B) dissection of the empyema. The thoracic cavity showed extensive adhesions which were carefully dissected bluntly, and the area was irrigated multiple times.

Due to the presence of a loculated pleural effusion and ineffective chest drainage, video-assisted thoracoscopic surgery (VATS) was performed on day 21. The thoracic cavity showed extensive adhesions that were carefully and bluntly dissected (Fig. 2A and B). The area was irrigated multiple times, and the wound was closed with the insertion of a chest drain.

On day 24, chest X-ray showed improvement in the right lung opacities (Fig. 1I). A follow-up CT scan also revealed a reduction in pus accumulation, though new pleural fluid was noted ventrally (Fig. 1J). The chest drain was removed due to obstruction by a hematoma, suggesting that further drainage would be difficult. Inflammatory markers in the blood gradually decreased, and after 6 weeks of intravenous antibiotics, the patient was transitioned to oral amoxicillin/clavulanic acid (750 mg/375 mg) plus amoxicillin 750 mg per day, as *Actinomyces* infections require prolonged treatment.

The patient was discharged on day 51 and followed up in the outpatient clinic. He completed an 11-month course of oral antibiotics, with a follow-up CT confirming full resolution of the empyema (Fig. 1K and L).

## 3 Discussion

We report a case of polymicrobial empyema in which the rare pathogen *Actinomyces georgiae* was identified. Despite initial treatment with antibiotics and chest drainage, the patient's condition did not improve, and successful treatment was achieved with VATS. The clinical course of this case provides valuable insights for clinicians managing similar infections.

Pulmonary actinomycosis is rare, with an incidence of approximately 1 case per 3,000,000 individuals annually. Although *A. israelii* and *A. gerencseriae* account for most actinomycosis cases, *A. graevenitzii* is the leading cause of pulmonary actinomycosis [6]. Empyema has been reported to be associated with *Actinomyces* species, including *A. meyeri*, *A. israelii*, *A. odontolyticus*, *A. naeslundii*, *A. turicensis*, and *A. viscosus*; however, to our knowledge, empyema caused by *A. georgiae* has not been previously reported [[7], [8], [9], [10], [11], [12]]. In this case, *A. georgiae* was identified by MALDI-TOF MS. A previous study reported that the number of *Actinomyces* species that have been isolated has increased since the introduction of MALDI-TOF MS [13]. Therefore, the identification of *A. georgiae* in this case may be attributed to significant advancements in microbiological diagnostic techniques, particularly the introduction of MALDI-TOF MS. Actinomycosis typically results from the aspiration of gastrointestinal secretions, inhalation of oropharyngeal particles, or secondary spread from the cervicofacial region or distant sites [1]. In this case, cavities were suspected as the portal of entry for the infection.

Antimicrobials are usually administered for an extended duration in pulmonary actinomycosis due to the infection's tendency to recur [1]. Initial treatment typically involves 2–6 weeks of intravenous therapy, followed by a transition to oral therapy upon significant clinical improvement. Total therapy duration is generally 6–12 months [6]. In a meta-analysis of 12 randomized controlled trials, intrapleural fibrinolytic therapy was found to be associated with a reduced need for surgical intervention and a decrease in overall treatment failure [14], though no studies have demonstrated a mortality benefit. The British Thoracic Society (BTS) and European Respiratory Society (ERS) guidelines recommend the use of intrapleural fibrinolytics as a potentially surgery-sparing option when chest tube drainage and antibiotics fail [15,16]. In this case, urokinase was withheld due to CT findings suggestive of hemorrhage; however, its administration might have prevented the need for surgery.

In empyema management, thoracoscopic surgery is indicated when antibiotic therapy, chest drainage, and fibrinolytics are ineffective [17]. BTS guidelines for pleural disease lack clear criteria for the timing of referral to surgery and the definition of "failed medical treatment". A retrospective analysis of over 4000 patients undergoing intervention for empyema found higher rates of readmission and reintervention in those treated with chest tubes than in those treated with VATS decortication or open decortication, suggesting that earlier definitive surgical intervention may benefit some patients [18]. However, the chest tube cohort was notably older and had higher rates of major medical comorbidities. These baseline differences were not adjusted for in the analysis, and may themselves have contributed to the poorer outcomes observed in this group. In a review comparing 28 cases of empyema caused by *Actinomyces* with 38 cases caused by other pathogens, empyema due to *Actinomyces* was more frequently associated with the need for thoracotomy (15.8 % vs. 7.9 %) [19]. We consulted the thoracic surgery department on day 17, after the patient developed fever and a CT scan revealed worsening pleural effusion. The patient underwent debridement via VATS and continued antimicrobial therapy, resulting in clinical improvement.

This case has a limitation. Although *Actinomyces* infection was suggested by Gram staining of the pleural fluid, which revealed elongated Gram-positive rods, and the presence of *A. georgiae* was confirmed by culture test, the presence of other pathogens indicates that *A. georgiae* might be a contributory organism rather than the sole cause of the empyema. Nevertheless, its identification was critical for determining the extended duration of antibiotic therapy, which differs from typical empyema management.

## 4 Conclusions

We encountered a case of empyema associated with *Actinomyces georgiae* that did not improve with antibiotics and chest drainage but showed significant improvement following combined treatment including surgical intervention. Empyema caused by *Actinomyces* is more likely to require surgical intervention, and long-term antimicrobial therapy is essential for effective management. Therefore, *Actinomyces* should be considered a potential causative agent of empyema, and a comprehensive treatment approach, including surgical intervention, is crucial for optimal outcomes.

## CRediT authorship contribution statement

**Senichi Fukuda:** Writing – original draft, Visualization, Investigation, Data curation. **Yuya Homma:** Writing – review & editing, Investigation, Data curation. **Hiroki Kawakami:** Writing – review & editing. **Soichiro Yamaji:** Writing – review & editing. **Yuki Sato:** Writing – review & editing. **Nao Sato:** Writing – review & editing. **Reina Idemitsu:** Writing – review & editing. **Taiki Kawai:** Writing – review & editing. **Naoki Inoshima:** Writing – review & editing. **Jun Hayashi:** Writing – review & editing. **Norihiko Kubota:** Writing – review & editing. **Tatsuya Nagai:** Writing – review & editing. **Ayumu Otsuki:** Writing – review & editing. **Hiroyuki Ito:** Writing – review & editing. **Hiroshi Sugimura:** Writing – review & editing. **Kei Nakashima:** Writing – review & editing, Supervision.

## Funding

This research did not receive any specific grant from funding agencies in the public, commercial, or not-for-profit sectors.

## Declaration of competing interest

The authors declare that they have no known competing financial interests or personal relationships that could have appeared to influence the work reported in this paper.
